# One-Step Ultrasound-Assisted Synchronous Extraction of Polysaccharides and Polyphenols from Blue Honeysuckle Berries: Structural Characteristics and Associated Bioactivities

**DOI:** 10.3390/foods15101691

**Published:** 2026-05-12

**Authors:** Runzhou Gao, Lujie Zhang, Junwei Huo, Xiaonan Sui, Yan Zhang

**Affiliations:** 1 College of Horticulture and Landscape Architecture, Northeast Agricultural University, Harbin 150030, China; 2 National-Local Joint Engineering Research Center for Development and Utilization of Small Fruits in Cold Regions, Northeast Agricultural University, Harbin 150030, China; 3 Heilongjiang Green Food Science Research Institute, Northeast Agricultural University, Harbin 150030, China; 4 College of Food Science, Northeast Agricultural University, Harbin 150030, China

**Keywords:** haskap, polysaccharides, bioactivities

## Abstract

Blue honeysuckle (*Lonicera caerulea* L.) berries are rich in bioactive compounds, yet their efficient utilization is limited by conventional single-component extraction methods. This study developed a one-step ultrasound-assisted synchronous extraction (OUE) method to simultaneously recover polysaccharides and polyphenols and evaluate their physicochemical properties and bioactivities. OUE-extracted polyphenols (OUEP) and polysaccharides (OUES) showed significantly stronger antioxidant activity, with higher DPPH and ABTS radical scavenging capacities than conventionally extracted polyphenols (MPP) and hot water extraction followed by alcohol polysaccharides (HPS). FTIR analysis indicated similar polysaccharide backbones for OUES and HPS, while SEM revealed a more porous microstructure in OUES, and rheological measurements showed higher apparent viscosity, suggesting improved macromolecular interactions and functional behavior in food systems. Additionally, OUEP displayed strong α-amylase and α-glucosidase inhibitory activities (IC_50_ = 25.85 ± 1.77 and 26.08 ± 0.11 mg/mL, respectively), highlighting their potential for glycemic control. These findings demonstrate that OUE not only enables efficient, simultaneous recovery of multiple bioactive components but also enhances their structural and functional properties, supporting the development of health-promoting food products and improving the utilization of blue honeysuckle berries.

## 1. Introduction

Blue honeysuckle (*Lonicera caerulea* L.) is a deciduous shrub in the Caprifoliaceae family. Owing to its strong cold tolerance, it is predominantly distributed across cold and temperate regions, including China, Russia, Japan, and England. Blue honeysuckle berries are rich in bioactive constituents, particularly anthocyanins, flavonoids, and vitamins, which collectively contribute to their pronounced antioxidant, antimicrobial, and glucose-regulating activities [1].

Berries mainly contain two major classes of phytochemicals: polysaccharides and polyphenols. Polysaccharides are bioactive macromolecules made up of multiple monosaccharide units. Found extensively in both plants and animals, they fulfill a variety of physiological functions. In the food industry, polysaccharides exhibit important functional properties, such as gelation, emulsification, and thickening. Moreover, these compounds display diverse bioactivities, notably antidiabetic, antitumor, and antioxidant effects [2]. Polyphenols are plant-derived bioactive compounds characterized by multiple hydroxyl groups on aromatic rings, which underpin their broad range of biological activities. Polyphenols, which are abundant in plant foods, help combat oxidative stress and inflammation, regulate lipid metabolism, and may reduce the risk of cardiovascular and metabolic disorders [3].

The bioactivities of plant-derived polysaccharides and polyphenols are strongly influenced by the extraction techniques employed. Conventional techniques for isolating these compounds mainly include solvent extraction, deep eutectic solvent extraction, and supercritical fluid extraction. Solvent extraction is widely used due to its simple equipment, operational convenience, and relatively high yields; however, it is constrained by long extraction times and high consumption of organic solvents [4]. In contrast, deep eutectic solvent extraction and supercritical fluid extraction are considered environmentally friendly and green alternatives, but their operational complexity and high cost limit their suitability for large-scale industrial production [5]. To improve extraction efficiency, auxiliary techniques such as ultrasonic-, enzymatic-, and microwave-assisted extraction are often applied. Ultrasonic-assisted extraction enhances mass transfer by disrupting plant cell walls through cavitation effects, thereby facilitating the release of target compounds [4]. Enzymatic-assisted extraction operates under mild conditions, reduces solvent usage, and improves selectivity and environmental compatibility. Microwave-assisted extraction enables rapid and uniform heating, significantly shortening extraction time and increasing extraction efficiency [5]. In recent years, combined extraction strategies integrating multiple physical or biochemical techniques have been increasingly explored to further improve extraction efficiency. For instance, ultrasound–microwave-assisted extraction integrates microwave heating with ultrasonic cavitation to enhance cell disruption and mass transfer. Similarly, deep eutectic solvent–ultrasound extraction has emerged as a promising green strategy, in which deep eutectic solvents disrupt hydrogen-bond networks in plant matrices while ultrasonic cavitation enhances mass transfer and cell wall disruption [6,7]. In comparison with enzymatic and microwave-assisted techniques, UAE is characterized by shorter extraction time, lower operating temperatures, and reliable reproducibility.

In conventional single-extraction processes, residual plant material is often discarded, leading to the loss of other valuable components such as polysaccharides. Therefore, developing integrated strategies capable of simultaneously and efficiently extracting and separating multiple bioactive compounds with distinct properties from a single raw material has become a key direction in the field of green extraction. One-step ultrasound-assisted synchronous extraction (OUE), derived from aqueous two-phase extraction (ATPE), has gained increasing attention due to its operational simplicity and high extraction efficiency [4]. Among ATPE systems, alcohol–salt aqueous two-phase systems offer notable advantages, including low viscosity, low cost, simple phase separation, minimal toxicity, and high extraction performance, supporting their wide application [3]. In general, the aqueous two-phase system (ATPS) enables separation based on the differential partitioning of solutes between two immiscible aqueous phases. By combining ultrasound with an alcohol–salt ATPS, OUE allows selective partitioning of compounds according to their polarity and molecular characteristics: hydrophobic polyphenols preferentially migrate to the alcohol-rich upper phase, while hydrophilic polysaccharides remain in the salt-rich lower phase [8]. Meanwhile, ultrasound promotes cell disruption and mass transfer, significantly shortening the extraction time [7].

Rich in polyphenols and anthocyanins with potent antioxidant and anti-inflammatory effects, the blue honeysuckle berry also contains bioactive polysaccharides for added health benefits. To date, the co-extraction of polyphenols and polysaccharides has been reported only for blackcurrant among small berry fruits, while the synergistic release patterns among the components of blue honeysuckle, as a complex matrix rich in anthocyanins and acidic polysaccharides, have not yet been revealed [3]. Current extraction strategies for blue honeysuckle berries primarily target individual bioactive components, resulting in high energy consumption, prolonged processing times, and substantial loss of high-value polysaccharide macromolecules [1]. Consequently, there remains a clear gap in developing an efficient, integrated extraction method that can simultaneously recover multiple bioactive fractions while preserving their structural integrity.

To address this gap, we developed a one-step ultrasound-assisted synchronous extraction (OUE) strategy based on ultrasound-assisted aqueous two-phase extraction (UA-ATPE). This approach not only disrupts the cell wall through ultrasonic treatment but also provides an optimal microenvironment for the released polysaccharides and polyphenols, enabling their simultaneous extraction from blue honeysuckle. This strategy improves raw material efficiency and contributes to the integrated utilization and functional food development of blue honeysuckle bioactives.

Hypothesis of this study: We hypothesized that the one-step ultrasound-assisted synchronous extraction (OUE) based on an ethanol–ammonium sulfate aqueous two-phase system could simultaneously recover polysaccharides and polyphenols from blue honeysuckle berries. More importantly, compared with conventional single-component extraction methods, OUE could qualitatively enrich more bioactive polyphenol components, thereby enhancing their bioactivities—particularly antioxidant and enzyme inhibitory properties.

To test this hypothesis, the objectives of this study were to: (1) compare the extraction yields of polyphenols and polysaccharides obtained by conventional individual extraction and simultaneous co-extraction; (2) evaluate the antioxidant activity and inhibitory effects of blue honeysuckle berry polyphenols and polysaccharides against tyrosinase, acetylcholinesterase, α-amylase, and α-glucosidase; (3) characterize the rheological properties, including apparent viscosity and dynamic viscoelasticity; and (4) investigate the structural features of polysaccharides using SEM and FTIR.

## 2. Materials and Methods

### 2.1. Chemicals

Ripe blue honeysuckle berries (berries with a blue–purple to dark purple color and white waxy bloom, harvested when approximately 80% of fruits on the plant reached this stage) were harvested from the experimental farm of Northeast Agricultural University (126°37′39″ E, 45°42′22″ N). After harvest, the fruits were stored at −20 °C until further use in subsequent experiments. Dialysis bags were purchased from Biotopped Co., Ltd. (Beijing, China). Tyrosinase (mushrooms) was obtained from Yuanye Bio-Technology Co., Ltd. (Shanghai, China). Corn starch was obtained from Solarbio Co., Ltd. (Beijing, China). Folin–Ciocalteu reagent was obtained from Bomei Co., Ltd. (Hefei, China). Alpha-amylase, α-glucosidase, 2,2′-azinobis-(3-ethylbenzthiazoline-6-sulphonate) (ABTS), 2,2-diphenyl-1-picrylhydrazyl (DPPH), gallic acid, gallocatechin, catechin, quercetin-3-rutinoside, astilbin, chlorogenic acid, naringenin, luteolin, procyanidin 2,4,6-tripyridyl-s-triazine (TPTZ), and 6-hydroxy-2,5,7,8-tetramethylchroman-2-carboxylic acid (Trolox) were purchased from Sigma-Aldrich Chemical Co. (St Louis, MO, USA). HPLC-grade acetonitrile was obtained from Honeywell Co., Ltd. (Charlotte, NC, USA). All other materials used in this study were analytically pure grade and purchased from Tianda Co., Ltd. (Tianjin, China).

### 2.2. Extraction of Blue Honeysuckle Berry Polyphenols

Blue honeysuckle berries were frozen at −80 °C for 24 h and lyophilized (VACO-2-8, Zirbus, Bad Grund, Germany) under vacuum (<20 Pa) at −80 °C for 48 h. The lyophilized berries were then ground into a fine powder in liquid nitrogen using a mortar and pestle. The lyophilized powder (10 g) was extracted with 100 mL of 80% methanol to obtain crude polyphenols. The suspension was shaken at 200 rpm for 4 h using a shaker (SHZ-C, BOXUN, Beijing, China) and then centrifuged to collect the supernatant (X4R Pro, Thermo Fisher Scientific, Waltham, MA, USA). The combined supernatants were concentrated by rotary evaporation (R-100, BUCHI, Flawil, Switzerland) at 40 °C and subsequently filtered through a 0.22 μm membrane (ANPEL, Shanghai, China). The polyphenol fraction obtained by 80% methanol extraction was designated as MPP.

### 2.3. Extraction of Blue Honeysuckle Berry Polysaccharides

One gram of blue honeysuckle berry powder was extracted with water at a solid-to-liquid ratio of 1:40 (90 °C, 2 h), according to the method described by Wang et al. [2], with slight modifications. The extract was then centrifuged, and the supernatant was subjected to ethanol precipitation. The precipitate was collected, redissolved in water, dialyzed (7 kDa cutoff, 72 h), centrifuged, freeze-dried, and redissolved. The polysaccharide fraction obtained by hot water extraction was designated as HPS.

### 2.4. Ultrasonic-Assisted Synchronous Extraction of Blue Honeysuckle Berry Polyphenols and Polysaccharides

The synchronous extraction of polyphenols and polysaccharides was performed according to Lin et al. with minor modifications [9]. Blue honeysuckle berry lyophilized powder (1.4 g) was extracted using an ethanol–ammonium sulfate solvent system, prepared by dissolving 11.72 g of ammonium sulfate in a mixture of 10 mL ethanol and 25 mL distilled water. The one-step ultrasound-assisted extraction (OUE) was performed at 80 °C using a probe-type ultrasound (350 W 20 kHz, 30 μm amplitude) for 43 min. After extraction, the mixture formed two distinct phases: the upper phase was a deep red clear solution, and the lower phase was an orange–yellow precipitate. The two phases were separated using a separatory funnel. Polyphenols in the upper phase were washed with ethanol (1:10, *v*/*v*) to remove precipitated ammonium sulfate, followed by centrifugation to collect the liquid fraction. The resulting polyphenol solution was concentrated and freeze-dried to obtain a powder. The lower phase was dialyzed against distilled water using a 7 kDa dialysis membrane for 72 h. Subsequently, the dialyzed solution was mixed with ethanol (1:10, *v*/*v*) and allowed to stand for 12 h to precipitate polysaccharides. The polyphenol and polysaccharide fractions obtained by OUE were designated as OUEP and OUES, respectively.

### 2.5. Measurement of Total Sugar Content (TSC)

TSC was measured using the phenol-sulfuric acid method [2]. Briefly, 1.0 mL of glucose standard or diluted blue honeysuckle sample was mixed with 1.0 mL deionized water and 1.0 mL 5% of (*w*/*v*) phenol. Then, 5.0 mL concentrated sulfuric acid was added and mixed. After cooling to 25 °C, absorbance was read at 490 nm, with deionized water as the blank.

### 2.6. Determination of Total Polyphenol Content (TPC), Total Anthocyanin Content (TAC) and Total Flavonoid Content (TFC)

TPC, TAC, and TFC were measured using a microplate reader (Epoch 2, BioTek, Winooski, VT, USA).

TPC was determined via the Folin–Ciocalteu method with slight modifications according to Zhang et al. A total of 90 μL of sample was mixed with 10 μL Folin–Ciocalteu reagent in a 96-well plate, incubated for 5 min in the dark, then 80 μL sodium carbonate (75 g/L) was added and incubated for 2 h before reading absorbance at 765 nm. Results were expressed as mg gallic acid equivalents (GAE)/g DW [1].

TAC was measured according to Zhang et al. A 20 μL sample was mixed with 180 μL KCl buffer (0.025 M, pH 1.0) or sodium acetate buffer (0.4 M, pH 4.5) in a 96-well plate, and absorbance was recorded at 510 and 700 nm. TAC was expressed as cyanidin-3-glucoside (C3G) equivalents [1]. TFC was determined using the aluminum chloride colorimetric method: 20 μL sample was mixed with 120 μL deionized water and 10 μL 5% sodium carbonate, incubated for 6 min, followed by 20 μL 10% AlCl_3_ (6 min) and 40 μL 4% NaOH (15 min). Absorbance was measured at 510 nm, and TFC was expressed as mg catechin equivalents (CAE)/g DW [10].

### 2.7. Determination of Antioxidant Capacity

#### 2.7.1. DPPH Assay

The DPPH assay was performed as described by Brand-Williams, Cuvelier, and Berset with minor modifications. Briefly, 5 μL of diluted sample was mixed with 195 μL of 60 μM DPPH in a 96-well plate and incubated in the dark at 25 °C for 2 h. Absorbance was read at 515 nm using a microplate reader [11].

#### 2.7.2. ABTS Assay

The ABTS assay was performed according to Re et al. [12]. ABTS (7 mM) and potassium persulfate (2.45 mM) were mixed in a 1:1 ratio and incubated in the dark for 12 h to generate ABTS+, which was diluted to an absorbance of 0.70 ± 0.02 at 734 nm. In a 96-well plate, 10 μL of sample was added to 190 μL ABTS+, incubated for 5 min, and absorbance was recorded at 734 nm.

#### 2.7.3. FRAP Assay

The FRAP assay was performed according to Zozio et al. [13]. FRAP reagent was freshly prepared by mixing TPTZ, FeCl_3_·6H_2_O, and acetate buffer (1:1:10, *v*/*v*/*v*). In a 96-well plate, 150 μL of FRAP reagent preheated to 37 °C was mixed with 10 μL sample and 30 μL deionized water. Absorbance at 593 nm was recorded for 30 min, and the 30 min value was used for quantification based on a FeSO_4_ standard curve.

### 2.8. Scanning Electron Microscopy (SEM) Analysis

The surface morphology of HPS and OUES samples was examined using a scanning electron microscope (SU8020, Hitachi, Tokyo, Japan) [2]. Dried polysaccharide samples were mounted on aluminum stubs using double-sided carbon tape. Images were captured at magnifications of 30× and 300× under an accelerating voltage of 3.0 kV.

### 2.9. Fourier Transform Infrared Spectroscopy (FTIR)

FTIR spectra of HPS and OUES were recorded using a Nicolet iN10 spectrometer (Thermo Fisher Scientific, Waltham, MA, USA) in the range of 4000–400 cm^−1^ with 64 scans at a resolution of 4 cm^−1^. Spectral data were analyzed using the instrument’s proprietary software [2].

### 2.10. Determination of α-Amylase and α-Glucosidase Inhibitory Activities

The α-amylase and α-glucosidase inhibitory activity was determined using a high-throughput turbidimetric method with slight modifications [14]. Twenty-five milliliters of phosphate-buffered saline (PBS, 0.01 M, pH 6.9) was prepared, and 0.5 g of corn starch was dissolved in the buffer. The mixture was then heated at 40 °C under constant stirring (400 rpm) for approximately 150 s until a semi-transparent colloidal state was achieved, indicating partial gelatinization of the starch. Solutions of α-amylase and α-glucosidase (4 mg/mL) were each prepared in PBS for subsequent use in enzymatic assays. Aliquots of gradient-diluted blue honeysuckle polyphenol and polysaccharide solutions (20 μL) were mixed with α-amylase and α-glucosidase solutions (0.2 mg/mL, 2 U/mL) in a 96-well microplate. Deionized water was used as the blank control. Acarbose (0–0.034 mg/mL) was used as a positive control. The mixtures were pre-incubated at 37 °C for 15 min in a microplate reader. Subsequently, 60 μL of pre-gelatinized corn starch solution was added to initiate the reaction; absorbance at 660 nm was recorded every 1 min for 2 h, yielding a total of 120 data points for each reaction. Different concentration ranges were applied for each fraction: MPP (0–36 mg/mL), OUEP (0–13 mg/mL), and HPS and OUES (0–10 mg/mL). All experiments were performed in triplicate. Based on enzyme inhibition kinetics, a nonlinear regression model was established to describe the relationship between the concentrations of haskap (Lonicera caerulea) fractions and their inhibitory activities against α-amylase and α-glucosidase. The half maximal inhibitory concentration (IC_50_) was used as a quantitative parameter to assess the inhibitory potency of each fraction. The corresponding equations are presented as follows:

$$\mathrm{AUC}=0.5+\frac{A_{2}+A_{n-1}}{A_{1}}+0.5\times \frac{A_{n}}{A_{1}}$$

$$\mathrm{Inhibition}\;\left(\%\right)=\frac{{\mathrm{AUC}}_{1}{-\mathrm{AUC}}_{\mathrm{CK}}}{{\mathrm{AUC}}_{1}}\times 100$$

where AUC represents the integrated area under the fluorescence quenching curve; A_1_, A_2_, …, A_n_ denote the absorbance values measured at each time point (1 < *n* < 121, n ∈ Z). AUC_1_ corresponds to the integrated area of the fluorescence quenching curve for the sample group, while AUC_CK represents that of the control group. The IC_50_ value was calculated using a nonlinear regression equation.

### 2.11. Tyrosinase Inhibitory Activity

The tyrosinase inhibitory activity was determined following the method of Yin et al. [15] with slight modifications. Briefly, 0.01 M PBS buffer (pH 6.9), 2.5 mM L-DOPA solution, and 3 U/mL tyrosinase solution were prepared. Then, 280 μL of the L-DOPA solution and 10 μL of the blackcurrant extract were added to a 96-well plate and pre-incubated at 30 °C for 5 min. Subsequently, 10 μL of tyrosinase solution was added, and the absorbance was measured at 475 nm using a microplate reader at 0 min and 15 min under 30 °C. Deionized water was used as the blank control. To eliminate potential interference from the intrinsic color of the extracts, background correction was performed at the measurement wavelength for each sample. The tyrosinase inhibition was calculated using the following formula:

Inhibition(%) = 1 − (A_2_ − A_1_)/(B_2_ − B_1_)] × 100%

where A_1_ and A_2_ represent the absorbance of the sample at 0 and 10 min, respectively, and B_1_ and B_2_ denote the absorbance of the blank at 0 and 10 min.

### 2.12. Apparent Viscosity of Polysaccharides

The rheological properties of HPS and OUES samples were measured using a shear rheometer (HAAKE RS6000, Thermo Fisher Scientific, Waltham, MA, USA) equipped with a parallel stainless-steel plate (P60 Ti L; 60 mm diameter, 0.50 mm gap). After freeze-drying, 10 mg of each HPS and OUES sample was accurately weighed and dissolved in 10 mL of pH 7 deionized water to prepare solutions of the same concentration. After equilibration at 25 °C, apparent viscosity was determined over a shear rate range of 0.1–100 s^−1^ [2].

### 2.13. Viscoelasticity of Polysaccharides

Oscillatory strain sweep tests were first performed at 10 Hz over a strain range of 0.1–100% to determine the linear viscoelastic region. After freeze-drying, 10 mg of each HPS and OUES sample was accurately weighed and dissolved in 10 mL of pH 7 deionized water to prepare solutions of the same concentration, which were then subjected to frequency sweep tests in the linear viscoelastic region at a fixed strain of 5% over a frequency range of 0.1–10 Hz, and the storage modulus (G′) and loss modulus (G″) were recorded [2].

### 2.14. Characterization and Quantification of Polyphenol Compounds Using HPLC-ESI-QTOF-MS^2^

Non-anthocyanin polyphenols were analyzed by HPLC-DAD and HPLC-ESI-QTOF-MS^2^ (AB Sciex, Framingham, CA, USA) using a C18 column (Luna, 5 μm, 250 mm × 4.6 mm, Phenomenex, Torrance, CA, USA). Detection wavelengths were set at 280 and 520 nm, respectively. Chromatographic separation was performed at 25 °C using water containing 60 mM formic acid (mobile phase A) and acetonitrile containing 5 mM ammonium acetate (mobile phase B) at a flow rate of 1 mL/min. The gradient elution program was as follows: 14–16.5% B (0–12.5 min), 16.5–25% B (12.5–17.5 min), 25–80% B (17.5–40 min), 80–50% B (40–55 min), 50–14% B (55–60 min), followed by equilibration at 14% B for 5 min [1]. Mass spectrometric analysis was conducted in negative ion mode using an electrospray ionization source, with full-scan acquisition ranges of *m*/*z* 100–2000 (MS^1^) and *m*/*z* 50–2000 (MS^2^). The capillary voltage was set at 4500 V, and the drying gas temperature was maintained at 550 °C.

### 2.15. Statistical Analysis

All experiments were performed using freshly prepared samples in three independent biological replicates, and the results are expressed as mean ± standard deviation (SD). Statistical significance was evaluated by one-way analysis of variance (ANOVA) at a 95% confidence level using SPSS 21. Pearson correlation analysis and principal component analysis (PCA) were conducted using Origin 2021.

## 3. Results and Discussion

### 3.1. Extraction Yield of Polyphenols and Polysaccharides

**Figure 1:**
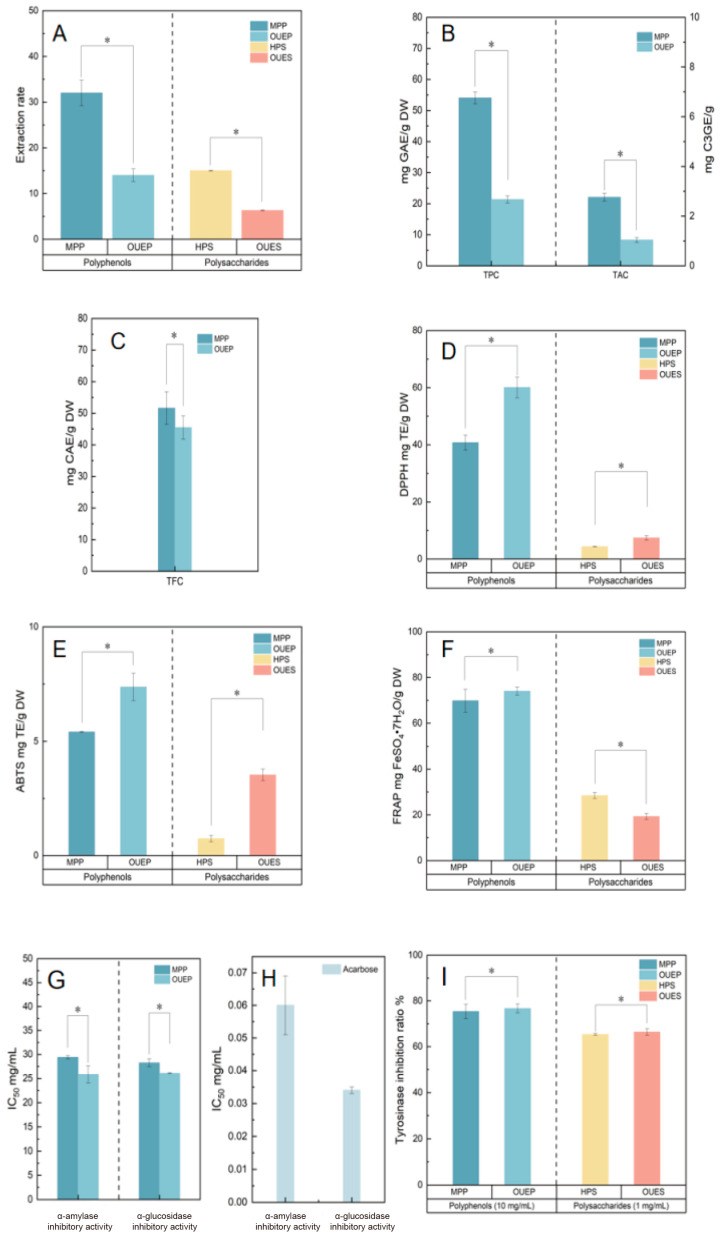
Extraction yields and bioactivities of polyphenols and polysaccharides obtained by different extraction methods. (**A**) Extraction yields of polyphenols and polysaccharides; (**B**) TPC and TAC; (**C**) TFC; (**D**) DPPH radical scavenging capacity; (**E**) ABTS radical scavenging capacity; (**F**) FRAP reducing capacity; (**G**) inhibitory activities of polyphenols and polysaccharides against α-amylase and α-glucosidase; (**H**) inhibitory activity of acarbose against α-amylase and α-glucosidase; (**I**) tyrosinase inhibitory activity of polyphenols and polysaccharides. MPP and OUEP represent polyphenols obtained by conventional solvent extraction and one-step ultrasound-assisted extraction, respectively. HPS and OUES are the polysaccharide fractions prepared by hot water extraction and the OUE method, respectively. Data are expressed as mean ± standard deviation (n = 3). Differences were analyzed by one-way ANOVA, and statistical significance was set at *p* < 0.05, with the asterisk (*) indicating a statistically significant difference.

The effects of conventional solvent extraction and OUE on the yields of polyphenols and polysaccharides were evaluated. As shown in Figure 1A, the extraction yields of MPP and OUEP were 32.0 ± 2.8% and 14.0 ± 1.4%, respectively, while those of HPS and OUES were 15.0 ± 0.1% and 6.3 ± 0.1%, respectively. OUE resulted in significantly reduced polyphenol and polysaccharide recoveries, with total yields decreasing by approximately 18% and 9%, respectively. The reduced yields may be attributed to ultrasonic treatment during the co-extraction process, which is absent in conventional solvent extraction. High-intensity ultrasonic pressure waves can induce partial degradation of polysaccharide macromolecules, thereby lowering their recoveries [16]. Similar trends have been reported in other plant materials. For example, polysaccharide yields from jujube obtained by ultrasonic-assisted extraction or UA-ATPE were approximately 60% of those achieved by conventional solvent extraction [17,18]. Likewise, polyphenol yields from olive leaves obtained using ultrasound-based or OUE methods were markedly lower than those obtained by traditional solvent extraction [19,20,21]. Despite the lower overall yields, OUE offers notable advantages. The ethanol/(NH_4_)_2_SO_4_ ATPS effectively reduces protein impurities, thereby improving polysaccharide purity [22]. Moreover, the synergistic effects of ultrasound and the ATPS promote cell wall disruption, facilitate the release and phase separation of target compounds, shorten processing time, reduce costs, and enable the simultaneous extraction of polyphenols and polysaccharides, thereby enhancing raw material utilization. From a practical perspective, the cost-effectiveness and scalability of the OUE process should also be considered. The integrated OUE strategy enables the simultaneous recovery of polyphenols and polysaccharides, potentially reducing solvent consumption, processing time, and operational costs, while also showing potential for industrial-scale application.

### 3.2. Comparison of TPC, TAC, and TFC

The phytochemical content differed significantly between the two methods (*p* < 0.05; Figure 1B,C). MPP yielded substantially higher total polyphenol content (TPC: 54.04 ± 1.88 mg GAE/g DW) and total anthocyanin content (TAC: 21.85 ± 0.30 mg C3GE/g DW) than OUEP. The total flavonoid content (TFC) was also higher in MPP (51.60 ± 5.13 mg CAE/g DW) than in OUEP (45.48 ± 3.67 mg CAE/g DW), though the difference was comparatively small. Similar reductions in polyphenols after ultrasonication have been reported [23]. Differences in both the content and composition of polyphenols extracted by different methods have also been reported. For example, in lotus rhizomes, 60% ethanol extraction yielded higher catechol content but lower levodopa content than UA-ATPE [4]. Similarly, OUEP exhibited lower polyphenol content and a distinct polyphenol profile compared with 80% methanol extraction in blue honeysuckle berries. These differences may arise from distinct extraction mechanisms. Conventional solvent extraction follows the “like dissolves like” principle, non-selectively solubilizing a broad range of polyphenols. In contrast, OUE relies on selective partitioning in the alcohol–salt aqueous two-phase system: moderately polar monomers (e.g., rutin, catechin) preferentially migrate to the ethanol-rich upper phase, while highly polar polyphenols tend to concentrate in the lower phase [8]. This explains the enrichment of rutin and catechin in OUEP and the higher levels of procyanidins and naringenin-glucoside in MPP, as confirmed by LC-MS (Table 1 and Table 2).

### 3.3. Antioxidant Capacity of Polyphenols and Polysaccharides from Blue Honeysuckle Berries

Interestingly, despite having a lower total polyphenol content, OUEP exhibited significantly stronger antioxidant capacity than MPP across all three assays (*p* < 0.05; Figure 1D–F). The DPPH scavenging capacity of OUEP (60.06 ± 3.66 mg TE/g DW) was 1.47-fold higher than that of MPP (40.76 ± 2.61 mg TE/g DW). Similarly, the ABTS scavenging capacity of OUEP (7.37 ± 0.60 mg TE/g DW) exceeded that of MPP (5.41 ± 0.02 mg TE/g DW) by 1.36-fold. The FRAP values followed the same trend, with OUEP (74.03 ± 1.78 mg FeSO_4_·7H_2_O/g DW) being 1.06-fold higher than MPP (69.82 ± 4.99 mg FeSO_4_·7H_2_O/g DW). A similar pattern was observed for the polysaccharide fractions (Figure 1D–F). OUES showed markedly higher DPPH scavenging capacity (7.40 ± 0.78 mg TE/g DW) than HPS (4.32 ± 0.09 mg TE/g DW), representing a 1.71-fold increase. The ABTS scavenging capacity of OUES (3.53 ± 0.25 mg TE/g DW) was 4.77-fold greater than that of HPS (0.74 ± 0.14 mg TE/g DW). However, the FRAP activity of OUES (19.30 ± 1.33 mg FeSO_4_·7H_2_O/g DW) was lower than that of HPS (28.44 ± 1.33 mg FeSO_4_·7H_2_O/g DW), being only 0.67 times that of the latter. Antioxidant capacity is a key biological property of plant extracts and is strongly influenced by extraction methods. Our findings are consistent with previous research demonstrating that advanced aqueous two-phase systems can yield fractions with high antioxidant potential. For instance, a study on blackcurrant fruits using a microwave-assisted aqueous two-phase system also successfully separated polysaccharide and polyphenol fractions, both of which demonstrated high DPPH radical scavenging activity values of 95–97% [3]. In lotus rhizomes, UA–ATPE-derived polyphenols exhibited stronger DPPH scavenging activity than those obtained by conventional ethanol extraction or ATPE alone [4]. Specifically, in the OUE system, we observed that antioxidant indices did not strictly follow the trend of total polyphenol content. This suggests that the bioactivity may be primarily driven by the qualitative polyphenol profile rather than the absolute quantity, although further studies (e.g., purification of individual compounds and activity testing) are needed to confirm this relationship. Previous studies have indeed reported that samples with lower total polyphenols can still exhibit strong antioxidant activity due to the presence of highly active polyphenol compounds [24]. Our LC-MS analysis provides supportive evidence for this observation: OUEP is significantly enriched in highly potent antioxidants compared to MPP. For instance, the content of rutin in OUEP (220.59 mg/100 g DW) was approximately 29.7-fold higher than in MPP (7.43 mg/100 g DW). Quercetin aglycone was also higher in OUEP (10.64 mg/100 g DW) than in MPP (6.72 mg/100 g DW), representing a 1.6-fold increase. Furthermore, (+)-catechin (108.66 mg/100 g DW) was exclusively detected in OUEP and absent in MPP. These compounds are well-known for their optimal structural features for radical scavenging [25]. The presence of these high-efficiency monomers, even at a lower total concentration, compensates for the lower TPC and results in superior overall bioactivity compared to the conventional extract (MPP), which may contain higher amounts of less active polyphenol fractions. Although limited information is available on the antioxidant activity of berry polysaccharides, our results indicate that OUES polysaccharides from blue honeysuckle berries possess superior DPPH and ABTS scavenging capacities compared with those obtained by hot water extraction. As shown in Figure 4D, polysaccharide yield correlated negatively with DPPH and ABTS, but positively with FRAP (r = −0.99, −0.96, 0.97). In addition, it is crucial to consider the potential for sonochemical degradation of the bioactive compounds themselves. We speculate that the mechanical shear forces generated during ultrasonic treatment may disrupt glycosidic bonds or hydrogen bonds in polysaccharides, leading to polysaccharide degradation, conformational changes, and alterations in antioxidant activity [26,27], such as molecular weight distribution, monosaccharide composition, uronic acid content, and degree of esterification. The proposed mechanism remains speculative and requires further verification.

### 3.4. α-Amylase and α-Glucosidase Inhibitory Activity from Blue Honeysuckle Berry

As shown in Figure 1G,H, the IC_50_ values for α-amylase inhibition by MPP, OUEP, and acarbose were 29.40 ± 0.34, 25.85 ± 1.77 and 0.06 ± 0.009 mg/mL, respectively, whereas polysaccharides from HPS and OUES showed no detectable inhibitory activity at 10 mg/mL. Similarly, the IC_50_ values of MPP, OUEP, and acarbose against α-glucosidase were 28.25 ± 0.81, 26.08 ± 0.11 and 0.034 ± 0.001 mg/mL, respectively, while no inhibition was observed for HPS or OUES polysaccharides at the same concentration. Previous studies have reported that the SPE-purified non-anthocyanin polyphenol fraction from blue honeysuckle (‘14-13-1’ cultivar) exhibited an IC_50_ value of 2.89 ± 0.62 mg/mL against α-amylase, which is higher than that observed in the present study. This discrepancy may be attributed to the solid-phase extraction (SPE) purification step applied in the previous work, which can effectively remove interfering matrix components and selectively enrich bioactive polyphenols, thereby enhancing the inhibitory activity. In contrast, the present study used crude polyphenol extracts without further purification [1]. Research on the inhibitory effects of co-extracted polyphenols and polysaccharides from small berries on α-amylase and α-glucosidase remains limited. In the present study, OUEPs exhibited significantly stronger inhibitory activities against both enzymes than MPP polyphenols (*p* < 0.05), whereas polysaccharides from either extraction method were inactive. The higher inhibitory activity of polyphenols may be attributed to their relatively planar molecular structures, which allow them to access the hydrophobic catalytic pockets of α-amylase and α-glucosidase. Their hydroxyl groups form hydrogen bonds with catalytic residues (e.g., Asp, Glu), while their aromatic rings contribute to hydrophobic interactions within the active site. These combined features—planarity, hydrogen bonding, and hydrophobic interactions—enhance inhibitory activity. In contrast, the complex three-dimensional conformations of polysaccharides likely impose steric hindrance, limiting such interactions [28]. To our knowledge, limited studies have reported the effects of co-extracted polyphenols and polysaccharides from blue honeysuckle berries on α-amylase and α-glucosidase activities. Differences in extraction methods led to marked variations in polyphenol composition between OUEP and MPP. LC-MS analysis revealed higher levels of neochlorogenic acid, quercetin-3-O-rutinoside, kaempferol, catechin, caffeic acid, quercetin, and quercetin-acetyl-hexoside in OUEP, compounds whose hydroxyl groups are known to interact with the active sites of α-amylase and α-glucosidase, thereby inhibiting enzymatic activity [28,29]. Thus, the stronger inhibitory effects of OUEP likely result from its higher content of these bioactive compounds. It should be noted that the IC_50_ values reported in this study were obtained using crude polyphenol extracts. The presence of non-active components (e.g., sugars, organic acids, and other matrix constituents) in the crude extracts likely dilutes the actual inhibitory potency, resulting in higher IC_50_ values. Future studies involving purification of the active polyphenol fractions are needed to determine their intrinsic enzyme inhibitory activities.

### 3.5. Tyrosinase Inhibitory Activity

As shown in Figure 1I, the tyrosinase inhibitory rates of MPP- and OUEP-derived polyphenols at 10 mg/mL were 75.37 ± 3.16% and 76.69 ± 1.95%, respectively. At 1 mg/mL, polysaccharides from HPS and OUES exhibited tyrosinase inhibitory rates of 65.34 ± 0.49% and 66.39 ± 1.45%, respectively. Research on the tyrosinase inhibitory activity of co-extracted polyphenols and polysaccharides from small berries remains limited; this study investigated such activities in co-extracts from blue honeysuckle berries. Tyrosinase inhibition did not differ significantly between polysaccharides obtained via the two extraction methods (*p* > 0.05). This may be attributed to their similar structural features, such as their comparable hydroxyl group content. Hydroxyl groups can chelate the copper ions in the tyrosinase active site or form hydrogen bonds with key amino acid residues, thereby inhibiting enzyme activity. In addition, polysaccharides may inhibit tyrosinase through multiple pathways, including substrate competition, copper chelation, and interference with melanogenesis-related signaling. The complex interplay of these mechanisms can lead to comparable overall inhibitory effects despite subtle structural variations [30].

Although dialysis with a 7 kDa membrane effectively removed most free low-molecular-weight compounds, including polyphenol substances, the complete removal of all phenolic compounds from complex natural preparations remains challenging. Trace amounts of phenolics may persist after dialysis via non-covalent interactions or physical entrapment, and these could enhance or synergize with polysaccharide activity. Thus, their potential contribution to the observed activity cannot be entirely excluded. To precisely determine the individual roles of polysaccharides versus associated phenolics, future research will focus on more advanced purification techniques and comprehensive quantification-activity comparisons. Likewise, despite marked differences in polyphenol composition between MPP and OUEP, their tyrosinase inhibitory activities did not differ significantly (*p* > 0.05). Tyrosinase inhibition by polyphenols is a complex process, often involving multiple inhibitors with varying potencies and diverse modes of action (e.g., competitive, non-competitive, mixed) [31]. Therefore, despite compositional differences, the net outcome of these intricate synergistic and antagonistic interactions can result in similar overall inhibitory activities between complex extracts.

### 3.6. FTIR Analysis

**Figure 2:**
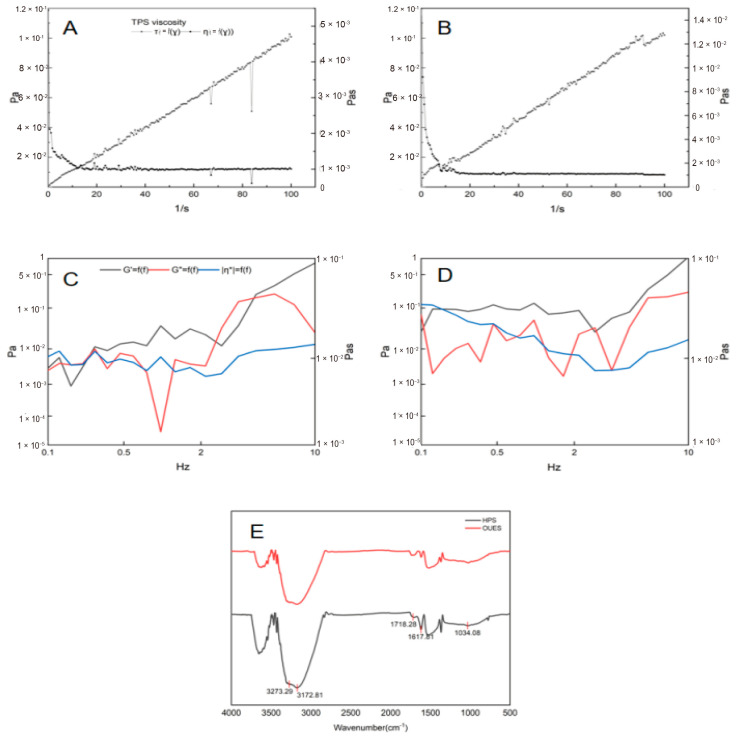
Rheological and structural characterization of polysaccharides obtained by two extraction methods. (**A**,**B**) Apparent viscosity of HPS and OUES, respectively; (**C**,**D**) viscoelastic properties of HPS and OUES, respectively; (**E**) FTIR spectra of polysaccharides obtained by the two extraction methods. HPS and OUES are the polysaccharide fractions prepared by hot water extraction and the OUE method, respectively.

The structural characteristics of polysaccharides obtained by the two extraction methods were analyzed by FTIR spectroscopy (Figure 2E). The FTIR spectra exhibited broad absorption bands at 3273 and 3172 cm^−1^, corresponding to O-H stretching vibrations, while the band at 1034 cm^−1^ was attributed to C-O stretching vibrations of primary alcohols, with differing intensities observed between HPS and OUES samples. In addition, absorption bands at 1718 and 1617 cm^−1^ were attributed to esterified (-COOR) and free carboxylate (-COO-) groups, respectively. Compared with HPS, OUES exhibited a larger peak area at 1718 cm^−1^ and a smaller peak at 1617 cm^−1^. Previous studies have shown that the degree of esterification is associated with polysaccharide viscosity, which may be attributed to enhanced hydrophobic interactions and hydrogen bonding [32]. It should be noted that the interpretation of the esterification degree based on FTIR is preliminary, and further analyses including monosaccharide composition and molecular weight distribution will be conducted in future studies to fully characterize the structural features of OUES and HPS.

### 3.7. SEM Analysis of Polysaccharides

**Figure 3:**
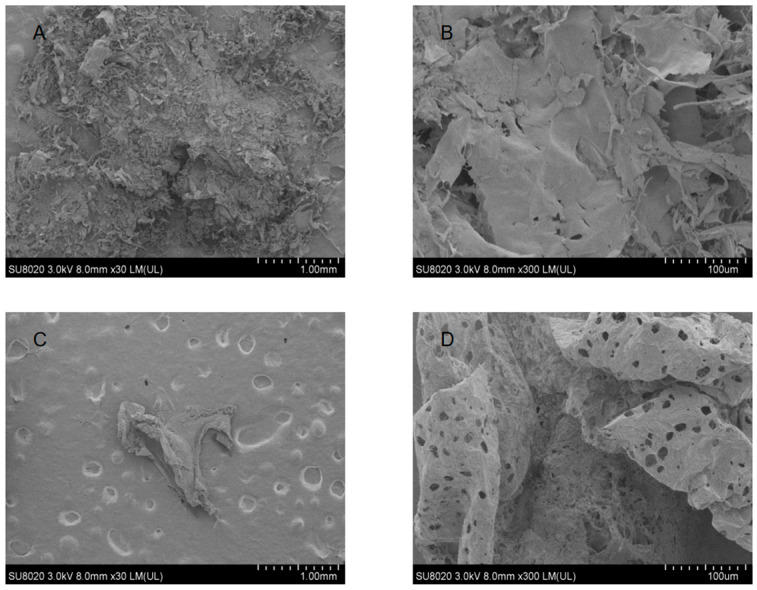
SEM micrographs of polysaccharides obtained by different extraction methods. (**A**,**B**) HPS; (**C**,**D**) OUES. HPS and OUES are the polysaccharide fractions prepared by hot water extraction and the OUE method, respectively.

The surface morphology of polysaccharides obtained using different extraction methods was examined by SEM at magnifications of 30× and 300× (Figure 3). Both samples predominantly exhibited flake-like structures. Notably, OUES-derived polysaccharides displayed a higher surface porosity than those obtained by HPS. Although studies on small berries remain limited, similar observations have been reported for Choerospondias axillaris, where ultrasound-extracted polysaccharides showed flake-like morphologies with substantially larger pore sizes than those extracted by hot water [2]. The increased porosity observed in OUES may be associated with cavitation and turbulent effects during ultrasound-assisted extraction, which can partially disrupt polysaccharide structures. Previous studies have suggested that ultrasound-induced disruption may result in a looser microstructure in polysaccharides. Such porous microstructures may contribute to the higher apparent viscosity observed in OUES, as increased surface area and structural openness can facilitate chain–chain interactions in solution. Nevertheless, a direct causal relationship between porosity and rheological properties was not established in this study and warrants further investigation [33].

### 3.8. Apparent Viscosity of Polysaccharides

As shown in Figure 2A,B, HPS and OUES solutions at the same concentration exhibited pronounced changes in apparent viscosity with increasing shear rates. Over the shear rate range of 0–100 s^−1^, the apparent viscosity of both polysaccharide solutions decreased as the shear rate increased, indicating typical shear-thinning behavior. A sharp decrease in viscosity was observed at shear rates below 20 s^−1^, whereas a more gradual decline occurred at higher shear rates. This behavior can be attributed to the disentanglement and alignment of polysaccharide chains under shear: at rest or low shear rates, extensive molecular entanglement leads to higher viscosity, while increasing shear disrupts these interactions, resulting in reduced viscosity [34]. Both HPS and OUES exhibited pseudoplastic flow behavior, a characteristic commonly observed in plant-derived polysaccharides, including those from blackcurrant [35]. Across the entire shear rate range, OUES consistently showed higher apparent viscosity than HPS, which may be associated with its higher degree of esterification. The apparent viscosity of polysaccharides depends on molecular size and other factors, the degree of methoxylation, carboxyl dissociation, and the number of carboxyl substituents [32]. These results suggest that OUES polysaccharides may exhibit enhanced thickening capacity under the tested conditions compared with HPS, underscoring their potential applicability as functional ingredients in food systems.

### 3.9. Viscoelasticity of Polysaccharides

Polysaccharides show viscoelasticity, which was evaluated using oscillatory frequency sweep tests. As shown in Figure 2C,D, within 0.1–10 Hz, the HPS sample showed crossover points of storage (G′) and loss (G″) moduli at 0.1387, 0.2552, and 2.525 Hz, while the OUES sample exhibited G′-G″ crossovers at 0.1103, 2.665, and 2.895 Hz. The crossover of G′ and G″ indicates a gel–sol transition, a behavior also observed in citrus pectin systems [36]. As frequency increased, high-frequency oscillations disrupted hydrogen bonding and hydrophobic interactions among polysaccharide chains, resulting in structural collapse and a transition to a viscous, liquid-like state [37]. These results demonstrate that both HPS and OUES polysaccharides possess favorable viscoelastic properties, which may contribute to improved flow behavior and swallowing characteristics in food and beverage applications.

### 3.10. Quantification and Qualification of Non-Anthocyanin Polyphenols

The characterization and quantification of polyphenols in MPP and OUEP were performed using HPLC-ESI-QTOF-MS^2^, and the related MS spectra along with quantitative data are summarized in Table 1 and Table 2.

Phenolic acids: Both MPP and OUEP were dominated by hydroxycinnamic acid derivatives, including dicaffeoylquinic acid, neochlorogenic acid, 3-O-caffeoylquinic acid, caffeic acid, 5-p-coumaroylquinic acid, and a trihydroxycinnamoylquinic acid isomer. Identification was based on fragment ions at *m*/*z* 191 (quinic acid moiety) and neutral losses corresponding to caffeoyl (162 Da) or p-coumaroyl (147 Da) groups. In contrast, OUEP contained a unique phenolic acid. Peak 12 had [M-H]^−^ 367, with a fragment loss of 174 Da (feruloylquinic acid anion), 176 Da (ferulic acid anion), and 18 Da (H_2_O). The ions at *m*/*z* 193, 191, and 173 correspond to 5-O-feruloylquinic acid. These compounds were identified by HR-MS^2^, and the identification process was as follows: [M-H]^−^ at *m*/*z* 515 with fragments 353, 191 was observed for dicaffeoylquinic acid [38]. Neochlorogenic acid ([M-H]^−^ 353) showed a characteristic fragment of 191 [39]. 3-O-Caffeoylquinic acid had a [M-H]^−^ at *m*/*z* 353 with a fragment at 191, while caffeic acid had a [M-H]^−^ at *m*/*z* 179 [1]. 5-p-Coumaroylquinic acid had a [M-H]^−^ at *m*/*z* 337 with fragments at 191 and 93 [38]. A trihydroxycinnamoylquinic acid isomer showed a [M-H]^−^ ion at *m*/*z* 371 with fragments at *m*/*z* 191. 5-O-Feruloylquinic acid, unique to OUEP, [M-H]^−^ 367 yielded fragments of 193 and 173 [40]. In MPP, dicaffeoylquinic acid was the predominant phenolic acid (109.04 ± 7.42 mg/100 g DW), whereas the trihydroxycinnamoylquinic acid isomer was present at the lowest level. In contrast, OUEP was characterized by a higher proportion of mono-caffeoylquinic acids, with 3-O-caffeoylquinic acid being the most abundant (105.08 ± 10.19 mg/100 g DW). These results suggest that OUE may favor the extraction of less polymerized hydroxycinnamate derivatives.

Flavanols: MPP mainly contained oligomeric proanthocyanidins, including B-type procyanidin dimers and trimers, as evidenced by characteristic fragment ions at *m*/*z* 289. In contrast, OUEP was enriched in flavan-3-ol monomers, with (+)-catechin and (−)-epicatechin as the main flavanols. (+)-Catechin was the most abundant (108.66 ± 3.35 mg/100 g DW). The mass spectrometric identification process was as follows. B-type procyanidin dimers and trimers, with [M-H]^−^ at *m*/*z* 577 and 865, were characterized by a fragment ion at *m*/*z* 289. (+)-Catechin and (−)-epicatechin both had [M-H]^−^ at 289, with (+)-catechin producing MS/MS signals at 245 and 123, and (−)-epicatechin yielding ions of *m*/*z* 245 and 125 [39].

Flavonols: Flavonols were mainly present as quercetin derivatives in both extracts. Multiple quercetin glycosides exhibited the aglycone ion at 301. MS identification was conducted as follows. Quercetin ([M-H]^−^ *m*/*z* 301) can form a series of glycosides by conjugation with different sugar moieties, including hexoside-pentoside (294 Da), vicianoside (294 Da), rutinoside (294 Da), glucoside (162 Da), rhamnosyl-hexoside (308 Da), robinobioside (308 Da), pentoside (322 Da), and acetyl-hexoside (203 Da). Among them, glucoside was unique to the MPP sample, whereas pentoside and acetyl-hexoside were unique to the OUEP sample. Isorhamnetin-3-O-rutinoside had [M-H]^−^ 623, with the aglycone isorhamnetin at 315 [39]. Kaempferol contained [M-H]^−^ 285 [41]. [M-H]^−^ 447 for kaempferol-3-O-glucoside, with aglycone kaempferol 285 [38]. In MPP, quercetin-3-O-vicianoside was the major flavonol (55.68 ± 1.52 mg/100 g DW), then quercetin-3-O-rhamnosyl-hexoside (40.8 ± 1.57 mg/100 g DW). Conversely, quercetin-3-O-rutinoside (rutin) predominated in OUEP (220.59 ± 1.32 mg/100 g DW), then quercetin-acetyl-hexoside (136.66 ± 0.19 mg/100 g DW). Overall, OUE markedly enhanced the extraction of quercetin-3-O-rutinoside and acetylated quercetin glycosides, whereas MPP exhibited greater structural diversity among quercetin derivatives.

Flavanone: Naringenin-7-O-glucoside ([M-H]^−^ 433) produced fragments of 271 and 151. The ion at 271 likely arises from cleavage of a glucose moiety (162 Da). This compound was detected in both MPP and OUEP extracts [38]. However, its content was substantially higher in MPP (247.51 ± 16.96 mg/100 g DW) than in OUEP (4.61 ± 0.29 mg/100 g DW), indicating that conventional solvent extraction preferentially retains flavanone glycosides.

Flavones: Luteolin-3-O-rutinoside and amentoflavone were detected in both MPP and OUEP extracts. Two flavones, luteolin-3-O-glucoside and eriodictyol-glucoside, were exclusively detected in MPP, whereas luteolin-O-hexoside-O-deoxyhexoside was uniquely identified in OUEP. Luteolin-3-O-rutinoside was the dominant flavone in both extracts, with a slightly higher content in OUEP (42.19 ± 1.25 mg/100 g DW) than in MPP (36.87 ± 3.17 mg/100 g DW). MS analysis was performed as follows. Luteolin-3-O-rutinoside, luteolin-3-O-glucoside, and Luteolin-O-hexoside-O-deoxyhexoside had [M-H]^−^ 593, 447, and 593, all yielding the luteolin aglycone at 285. Amentoflavone gave a [M-H]^−^ ion at 537 [1]. Eriodictyol-glucoside had [M-H]^−^ 449, yielding the eriodictyol aglycone at 287 and 151 [42].

Other compounds: Additionally, organic acids (malic acid and quinic acid) were detected in both extracts, while esculin, aromadendrin hexoside and methyl dicaffeoylquinate were unique to MPP. Aromadendrin hexoside was the most abundant compound in MPP (95.88 ± 6.66 mg/100 g DW). Hydroxytyrosol was exclusively detected in OUEP, albeit at low levels. MS identification was conducted as follows. Malic acid and quinic acid showed [M-H]^−^ 133 and 191, respectively [39]. Esculin had [M-H]^−^ 339, yielding characteristic fragments 177 and 133 [43]. Aromadendrin hexoside ([M-H]^−^ 449) gave the aromadendrin aglycone at 287 [44]. Methyl dicaffeoylquinate ([M-H]^−^ 529) gave a diagnostic fragment at 367 [45]. Hydroxytyrosol contained [M-H]^−^ 153 [46].

Overall comparison: In total, 30 compounds were identified in MPP and 25 compounds in OUEP. MPP exhibited higher compositional diversity, particularly in flavonols and proanthocyanidins. Naringenin-7-O-glucoside was the most abundant compound in MPP. In contrast, OUEP contained higher levels of specific bioactive compounds, notably quercetin-3-O-rutinoside (rutin), while quercetin derivatives exhibited the greatest structural diversity. As OUE has rarely been applied to blue honeysuckle berries, relevant studies are limited. Previous research on raspberry polyphenols showed that higher rutin content is associated with stronger DPPH scavenging activity, suggesting that the enhanced DPPH activity of OUEP may be related to its higher rutin content [47]. These differences can be attributed to the distinct extraction mechanisms, with OUE selectively enhancing the release of low-molecular-weight polyphenols while reducing the extraction of highly polymerized compounds. In addition, MPP and OUEP were obtained using different solvent and phase systems, which may influence the stability of certain polyphenol compounds and thereby affect their relative abundances. Future studies using spiking recovery or stability tests are needed to further verify the effects of extraction conditions on target compounds.

### 3.11. Correlation Analysis

**Figure 4:**
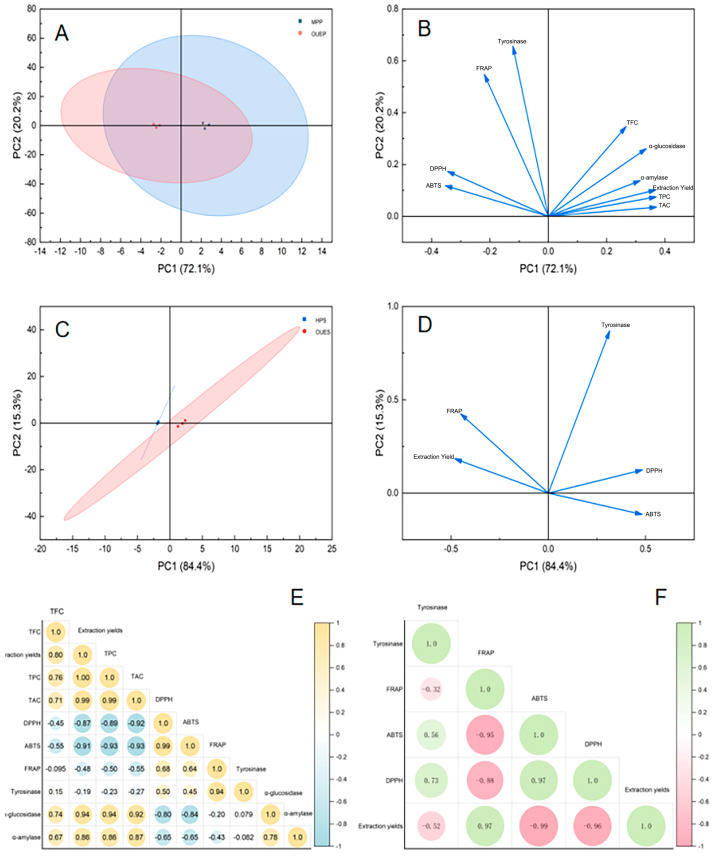
Multivariate analysis of polyphenols and polysaccharides from blue honeysuckle. (**A**,**B**) PCA score plots showing the associations of polyphenol samples obtained by two extraction methods with principal component 1 (PC1) and principal component 2 (PC2); (**C**,**D**) PCA score plots showing the associations of polysaccharide samples obtained by two extraction methods with PC1 and PC2; (**E**,**F**) Pearson correlation analysis between bioactivity parameters (ABTS, DPPH, FRAP, α-amylase, α-glucosidase, and tyrosinase inhibitory activities) and TPC, TAC, TFC, and TPAC for MPP and OUEP (**E**) and for HPS and OUES (**F**). MPP and OUEP represent polyphenols obtained by conventional solvent extraction and one-step ultrasound-assisted extraction, respectively. HPS and OUES are the polysaccharide fractions prepared by hot water extraction and the OUE method, respectively.

To visualize the overall differences between the extracts produced by the two methods, principal component analysis (PCA) was employed as an exploratory data visualization tool. As shown in the score plot (Figure 4A,B), the samples clustered into two distinct groups based on the extraction method, confirming a systematic difference between the OUE and conventional approaches. The loading plot reveals that the conventional method (MPP) is strongly correlated with higher yields and TPC, TFC and TAC. In contrast, the OUE method is closely associated with higher specific bioactivities, such as antioxidant capacity (DPPH, ABTS) and enzyme inhibition (α-amylase, α-glucosidase). While the limited number of groups warrants caution against overinterpretation, this visualization tentatively suggests that the two methods produce extracts with fundamentally different quantitative and qualitative profiles. This finding prompts a deeper mechanistic discussion, although a larger sample size would be needed to confirm this observation.

### 3.12. Limitations of the Study

Several limitations exist in this study. The chemical composition of blue honeysuckle berries may vary with cultivar, origin, climate, and fruit maturity, and batch variability cannot be completely excluded despite controlled harvesting. Therefore, the findings reported here may not be directly generalizable to berries from other sources or harvest seasons. In addition, only a single batch of fruits was used, which is a major limitation, as it remains unknown whether the observed advantages of OUE (e.g., enrichment of rutin, higher antioxidant activity) are reproducible across different batches. Future studies should validate the robustness of the results using multiple batches from different years or locations. The stability of the extracts under different storage conditions was not evaluated, limiting understanding of their shelf life and practical application. This is particularly important for industrial applications, where extracts may need to be stored for extended periods before use. Regarding scalability, challenges such as equipment scale-up (e.g., large-volume ultrasonic reactors), energy consumption, and efficient recovery and recycling of ethanol and ammonium sulfate from the aqueous two-phase system need to be addressed before industrial application.

## 4. Conclusions

This study systematically compared OUE with conventional solvent extraction for the simultaneous recovery of polyphenols and polysaccharides from blue honeysuckle berries. Although conventional extraction yielded slightly higher amounts, OUE significantly improved the biological activities of the extracts. OUE-derived polyphenols and polysaccharides exhibited enhanced antioxidant capacity, and the polyphenol fraction showed strong α-amylase and α-glucosidase inhibitory activities. Nevertheless, these findings are preliminary, as factors such as batch-to-batch consistency and large-scale production feasibility remain to be investigated. From an industrial perspective, the OUE strategy offers a more cost-effective and sustainable approach by integrating extraction and primary separation into a single step, reducing processing time, energy consumption, and solvent use. However, challenges such as equipment scale-up, energy consumption, and solvent recovery need to be addressed before industrial application. It enables the conversion of a single raw material into multiple value-added products (a polyphenol-rich liquid and a polysaccharide-rich solid), supporting circular bioeconomy principles. The resulting extracts, with enhanced functional properties, show potential as multifunctional clean-label ingredients for functional food and nutraceutical applications.

Overall, OUE represents an efficient and green strategy for the comprehensive valorization of blue honeysuckle berries. Future studies should further elucidate the mechanism of OUE and the polyphenol–polysaccharide interactions, while also validating the in vivo efficacy and practical application performance of the extracts in real food systems. Nevertheless, the findings are preliminary, and limitations such as batch variability, a lack of external validation, and scalability constraints should be acknowledged. Additional limitations include the lack of appropriate control experiments, limited structural characterization of polysaccharides (molecular weight distribution and monosaccharide composition were not analyzed), and the relatively high IC_50_ values of the crude extracts, which should be interpreted with caution.

## Figures and Tables

**Table 1:** Identification of non-anthocyanin polyphenols obtained by conventional solvent extraction (MPP). Data are expressed as mean ± standard deviation (n = 3).

Peak	RT (min)	Chemical Formula	MW	MS Expected Value ([M-H]^−^) (*m*/*z*)	MS Actual Value ([M-H]^−^) (*m*/*z*)	MS^2^ (*m*/*z*)	Error (ppm)	Tentative Identification	λmax (nm)	Content (mg/100 g DW)
1	2.63	C_16_H_20_O_10_	372	371.1189788	371.1191185	191.0560	−0.37	Trihydroxycinnamoylquinic acid (isomer)	195	3.73 ± 0.40
2	2.95	C_4_H_6_O_5_	134	133.0145913	133.0145415	115.0034/71.0133	0.37	Malic acid	223	14.73 ± 4.68
3	3.76	C_7_H_12_O_6_	192	191.0202568	191.0202641	111.0078/87.0080	−0.03	Quinic acid	203	5.12 ± 0.04
4	5.43	C_25_H_24_O_12_	516	515.1398517	515.140648	191.0565/353.0862	1.54	Dicaffeoylquinic acid	197	109.04 ± 7.42
5	7.78	C_16_H_18_O_9_	354	353.0877273	353.0877965	191.0565	−0.19	Neochlorogenic acid	231	40.48 ± 5.55
6	8.75	C_27_H_30_O_9_	340	339.0721001	339.0718468	177.0194/133.0283	0.74	Esculin	235	1.61 ± 0.06
7	12.37	C_30_H_24_O_12_	578	577.1351404	577.1347971	451.1003/425.0871/407.0783/289.0742/125.0244	0.59	Procyanidin dimer 1 (B-type)	280	5.96 ± 0.72
9	17.17	C_16_H_18_O_9_	354	353.0874987	353.0875999	191.0565	−0.28	3-O-caffeoylquinic acid	229	40.48 ± 2.20
10	17.48	C_45_H_36_O_18_	866	865.1998784	865.1986016	695.1521/577.1445/289.0752	1.47	Procyanidin trimer (B-type)	237	128.96 ± 36.04
11	18.5	C_9_H_8_O_4_	180	179.0354283	179.035577	135.0450/134.0378	−0.83	Caffeic acid	240	4.09 ± 1.18
12	19.82	C_30_H_24_O_8_	338	337.0928254	337.0929188	191.0565/93.0341	−0.27	5-p-Coumaroylquinic acid	236	15.35 ± 4.27
13	19.91	C_21_H_22_O_11_	450	449.1089939	449.1088108	287.0569//269.0464/125.0238	0.40	Aromadendrin hexoside	235	95.88 ± 6.66
14	22.02	C_26_H_32_O_18_	596	595.1347412	596.1345103	301.0364	0.38	Quercetin-3-hexoside-pentoside	258	12.79 ± 1.69
15	22.21	C_26_H_28_O_16_	596	595.131002	595.1310068	301.0354/300.0272/255.0305	−0.01	Quercetin-3-O-vicianoside	257	55.68 ± 1.52
16	22.46	C_27_H_30_O_16_	610	609.1454291	609.1452904	447.0947/301.0349/300.0288	0.22	Quercetin-3-O-rutinoside (Rutin)	241	7.43 ± 2.34
17	22.75	C_21_H_20_O_12_	464	463.088214	463.0882	301.0375/151.0038	0.17	Quercetin 3-O-glucoside	242	17.13 ± 1.07
18	23.14	C_27_H_30_O_16_	610	609.1461914	609.1464389	301.0358/300.0278	−0.40	Quercetin-3-O-rhamnosiy-hexoside	255	40.8 ± 1.57
19	23.57	C_21_H_20_O_11_	448	447.1833588	447.1839039	285.04241	−1.21	Luteolin 3-O-glucoside	242	7.73 ± 2.39
20	23.82	C_30_H_26_O_12_	578	577.1356416	577.1354971	407.0785/289.0730/245.0818/125.0247	0.25	Procyanidin dimer 2 (B-type)	252	31.88 ± 2.06
21	24.32	C_27_H_30_O_16_	594	593.0454198	593.0435529	285.0422	3.14	Luteolin 3-O-rutinoside	243	36.87 ± 13.17
22	24.67	C_28_H_32_O_16_	624	623.2912991	623.2922275	315.0517/314.0439	1.48	Isorhamnetin-3-O-rutinoside	242	1.57 ± 0.33
23	24.71	C_20_H_20_O_12_	434	433.0775242	433.0775155	301.0374	0.02	Quercetin-pentoside	243	3.48 ± 0.55
24	25.02	C_23_H_25_O_17_	506	505.0983919	505.09847	301.0366/300.0295	−0.15	Quercetin- acetyl-hexoside	242	10.50 ± 0.85
25	25.7	C_21_H_22_O_10_	434	433.0775242	433.0775155	271.0626/151.0047	0.02	Naringenin-glucoside	245	247.51 ± 16.96
26	26.18	C_21_H_22_O_11_	450	449.2757226	449.2748188	287.0571/151.0046	2.01	Eriodictyol-glucoside	243	20.56 ± 2.81
27	28.01	C_27_H_30_O_16_	610	609.1263929	609.1249	301.0347/300.0281/271.0243	0.28	Quercetin-3-O-robinobioside	252	2.12 ± 0.47
28	29	C_26_H_26_O_12_	530	529.1350635	529.1351779	367.1035/349.0961	−0.21	Methyl dicaffeoylquinate	252	2.02 ± 0.18
29	30.34	C_15_H_10_O_6_	286	285.0408552	285.0409159	151.0005/133.0294	−0.21	Kaempferol	244	1.04 ± 0.07
30	30.7	C_15_H_10_O_7_	302	301.1650877	301.1649666	151.004	0.40	Quercetin	243	6.72 ± 1.32
31	33.27	C_30_H_18_O_10_	538	537.0826325	537.0826733	375.0521	−0.07	Amentoflavone	237	1.48 ± 0.28

**Table 2:** Identification of non-anthocyanin polyphenols obtained by one-step ultrasound-assisted synchronous extraction (OUEP). Data are expressed as mean ± standard deviation (n = 3).

Peak	Rt (min)	Chemical Formula	MW	MS Expected Value ([M-H]^−^) (*m*/*z*)	MS Actual Value ([M-H]^−^) (*m*/*z*)	MS^2^ (*m*/*z*)	Error (ppm)	Tentative Identification	λmax (nm)	Content (mg/100 g DW)
1	2.62	C_16_H_20_O_10_	372	371.1190875	371.1190443	191.0553	0.11	Trihydroxycinnamoylquinic acid (isomer)	200	0.531 ± 0.013
2	2.97	C_4_H_6_O_5_	134	133.0146622	133.0145645	115.0032	0.73	Malic acid	223	1.05 ± 0.08
3	3.34	C_7_H_12_O_6_	192	191.019925	191.0199711	111.0073/87.0080	−0.24	Quinic acid	226	15.81 ± 0.37
4	5.39	C_25_H_24_O_12_	516	515.1413637	515.1406491	353.0891/191.0562	1.38	Dicaffeoylquinic acid	227	4.86 ± 0.49
5	8.02	C_16_H_18_O_9_	354	353.0878269	353.0878273	191.0564	−0.01	Neochlorogenic acid	241	12.81 ± 0.35
6	8.8	C_8_H_10_O_3_	154	153.0196168	153.0194362	109.0295	1.18	Hydroxytyrosol	238	0.49 ± 0.001
7	14.01	C_15_H_14_O_6_	290	289.0718516	289.0718516	245.0832/123.0444	0.01	(+)-catechin	243/ 303	108.66 ± 3.35
8	17.15	C_16_H_18_O_9_	354	353.1816654	353.1814213	191.0565	0.6	3-O-caffeoylquinic acid	197	105.08 ± 10.19
9	17.97	C_15_H_14_O_6_	290	289.8842784	289.8843075	245.5973/125.8709	−0.10	(−)-Epicatechin	244	5.34 ± 0.86
10	18.39	C_9_H_8_O_4_	180	179.8408695	179.8412408	135.0433/134.0375	−2.06	Caffeic acid	249	8.11 ± 0.24
11	19.78	C_30_H_24_O_8_	338	337.0930711	337.0932776	191.0578	−0.61	5-p-Coumaroylquinic acid	250	2.50 ± 0.06
12	20.95	C_17_H_20_O_9_	368	367.1028714	367.1032739	193.0350/191.0543/173.0392	−1.09	5-O-Feruloylquinic acid	234	1.27 ± 0.02
13	22.12	C_26_H_32_O_18_	596	595.0631226	595.0634754	301.0357/300.0277	−0.59	Quercetin-3-hexoside-pentoside	252	16.75 ± 1.62
14	22.7	C_26_H_28_O_16_	596	595.1304933	595.1305037	301.0385	−0.01	Quercetin-3-O-vicianoside	252	13.65 ± 0.90
15	23.11	C_27_H_30_O_16_	610	609.1466851	609.1466	301.0376/300.0291/271.0272	−0.44	Quercetin-3-O-rutinoside (Rutin)	240	220.59 ± 1.32
16	23.11	C_27_H_30_O_16_	610	609.932464	609.9327098	301.0371/300.0287	−0.40	Quercetin-3-O-rhamnosiy-hexoside	244	21.15 ± 0.66
17	23.66	C_22_H_22_O_11_	448	447.1504112	447.1503812	285.0414	0.06	Kaempferol glucoside	246	19.05 ± 0.05
18	23.98	C_27_H_30_O_16_	594	593.0440987	593.0466065	285.0409	4.22	Luteolin 3-O-rutinoside	240	42.19 ± 1.25
19	24.34	C_26_H_28_O_16_	594	593.1517263	593.1517272	285.0409	−0.01	Luteolin-O-hexose-O-deoxyhexoside	252	16.80 ± 2.48
20	24.44	C_28_H_32_O_16_	624	623.1628081	623.163	315.0507/314.0448/300.0276	0.47	Quercetin-pentoside	240	2.67 ± 2.89
21	25.78	C_23_H_25_O_17_	506	505.0986064	505.0988435	301.0350/300.0278	−0.46	Quercetin- acetyl -hexoside	246	136.66 ± 0.19
22	26.15	C_21_H_22_O_10_	434	433.1126553	433.1130221	271.0623/151.0032	−0.84	Naringenin-glucoside	235	4.61 ± 0.29
23	30.34	C_15_H_10_O_7_	302	301.0357545	301.0359038	301.0366	−0.49	Quercetin	241	10.64 ± 0.32
24	30.44	C_15_H_10_O_6_	286	285.0407026	285.0406991	151.0056/133.0289	0.01	Kaempferol	241	5.59 ± 0.01
25	33.28	C_30_H_18_O_10_	538	538.0840153	538.0827754	375.0532	2.30	Amentoflavone	242	3.14 ± 0.23

## Data Availability

The original contributions presented in this study are included in the article. Further inquiries can be directed to the corresponding author.
